# Single‐Cell Dissection of Tumor‐Infiltrating Lymphocytes Reveals Cellular Architecture Predictive of Therapeutic Efficacy in Acral Melanoma

**DOI:** 10.1002/advs.202521555

**Published:** 2026-01-04

**Authors:** Chao Zhang, Wanyi Xiao, Hongru Shen, Fenge Li, Ting Li, Weihong Zhang, Haotian Liu, Ziwei Gao, Hongyu Wang, Xiubao Ren, Kexin Chen, Xiangchun Li, Jilong Yang

**Affiliations:** ^1^ Department of Bone and Soft Tissue Tumors National Clinical Research Center for Cancer Tianjin Medical University Cancer Institute and Hospital Tianjin China; ^2^ Tianjin's Clinical Research Center for Cancer Tianjin China; ^3^ State Key Laboratory of Druggability Evaluation and Systematic Translational Medicine Tianjin China; ^4^ Department of Epidemiology and Biostatistics Key Laboratory of Molecular Cancer Epidemiology of Tianjin Tianjin Medical University Cancer Institute and Hospital Tianjin China; ^5^ Cancer Diagnosis and Treatment Center Tianjin Union Medical Center The First Affiliated Hospital of Nankai University Tianjin China; ^6^ Department of Immunology National Clinical Research Center for Cancer Tianjin Medical University Cancer Institute and Hospital Tianjin China

**Keywords:** acral melanoma, Clonal expansion, scRNA‐seq & scTCR‐seq, TEX_int, Tfh, TIL therapy

## Abstract

Acral melanoma (AM), the predominant melanoma subtype in Asia, responds poorly to immune checkpoint inhibitors, representing a critical unmet medical need. The efficacy of tumor‐infiltrating lymphocyte (TIL) therapy in this population is unknown. An Investigator‐Initiated Trial evaluates autologous TIL therapy (LM‐103) in four Chinese patients with advanced AM, achieving a 75% disease control rate (DCR) and a 25% objective response rate (ORR), including one durable complete response. To define the determinants of response, we performed integrated single‐cell RNA and T‐cell receptor sequencing on infused TIL products, tumors, and longitudinal peripheral blood. Responders' infused products were significantly enriched for T follicular helper (Tfh) and intermediate exhausted (TEX_int) CD8⁺ T cells, which mediated robust cell‐cell signaling networks (e.g., CD40, FASLG). In contrast, the non‐responder's product was dominated by terminally exhausted (TEX_term) cells. Clonal tracking revealed that these Tfh and TEX_int subsets possessed higher clonality, and in the complete responder, a dominant clone originating from the TEX_int population persisted systemically by differentiating into a progenitor‐like (TEX_prog) state. These findings demonstrate that TIL therapy is clinically active in AM and that durable response is mechanistically linked to the infusion and persistence of Tfh and TEX_int subsets, defining a key cellular and clonal architecture for therapeutic success.

## Graphical abstract

Autologous tumor‐infiltrating lymphocyte (TIL) therapy shows promising efficacy in acral melanoma, yet determinants of durable response remain unclear. By integrating single‐cell transcriptomics and TCR sequencing, this study reveals that TIL products enriched for T follicular helper and intermediate exhausted T cells establish persistent clonal immunity, providing mechanistic insight into effective adoptive cell therapy.

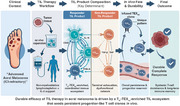

## 1 Introduction

Malignant melanoma represents a formidable global health challenge, with distinct epidemiological and biological features in different populations. In contrast to Caucasian populations, where sun‐exposed cutaneous melanoma is most common, acral melanoma—arising on the non‐hair‐bearing skin of palms, soles, and nail beds—is the predominant subtype in Asian populations, including in China [1, 2]. This distinction is not merely geographic; acral melanomas possess a unique biology characterized by a lower tumor mutational burden, different driver mutations, and a distinct tumor immune microenvironment, rendering them notoriously resistant to standard immune checkpoint inhibitors (ICIs) [3, 4, 5]. Consequently, objective response rates to anti‐PD‐1 therapy in this patient population are often disappointingly low, creating a critical unmet medical need for effective therapeutic strategies [6].

Adoptive cell therapy (ACT) using autologous tumor‐infiltrating lymphocytes (TILs) has emerged as a powerful modality capable of mediating durable tumor regression in patients with advanced melanoma [7, 8, 9, 10]. By isolating, expanding ex vivo, and reinfusing T cells that have naturally infiltrated a patient's tumor, TIL therapy delivers a personalized, polyclonal, and tumor‐reactive T cell repertoire. The clinical efficacy of this approach is well‐established in Western cohorts with cutaneous melanoma, where TIL products like lifileucel have achieved objective response rates of over 30% in heavily pretreated patients who have progressed on ICIs [11, 12]. A recent phase 3 trial further solidified its role, demonstrating superior progression‐free survival for TIL therapy compared to ipilimumab in a similar refractory setting [13].

Despite this progress, the efficacy of TIL therapy in acral melanoma remains largely unexplored, and the specific immunological features that dictate response versus resistance are poorly understood. The unique biology of acral melanoma raises a critical question: can TIL therapy overcome the intrinsic resistance of these tumors, and if so, what cellular and clonal characteristics of the infused T cells are required for success? Answering this question is essential for optimizing ACT and extending its benefits to a broader patient population.

Here, we report the results of an Investigator‐Initiated Trial (IIT) of autologous TIL therapy (LM‐103) in Chinese patients with advanced acral melanoma. We demonstrate that this therapy is clinically active, capable of inducing a durable complete response. Through integrated single‐cell RNA and T cell receptor sequencing of tumors, infused TIL products, and longitudinal peripheral blood samples, we uncover the immunological determinants of therapeutic success. We reveal that clinical response is strongly associated with the enrichment of T follicular helper (Tfh) and intermediate exhausted (TEX_int) T cells in the infused product. Furthermore, we show that durable immunity is driven by the clonal architecture of these subsets and the establishment of a persistent, self‐renewing progenitor‐like (TEX_prog) T cell reservoir in the circulation, providing a mechanistic blueprint for effective TIL therapy in this challenging disease.

## 2 Results

### 2.1 Autologous TIL Therapy Induces Durable Clinical Responses in Advanced Acral Melanoma

**FIGURE 1:**
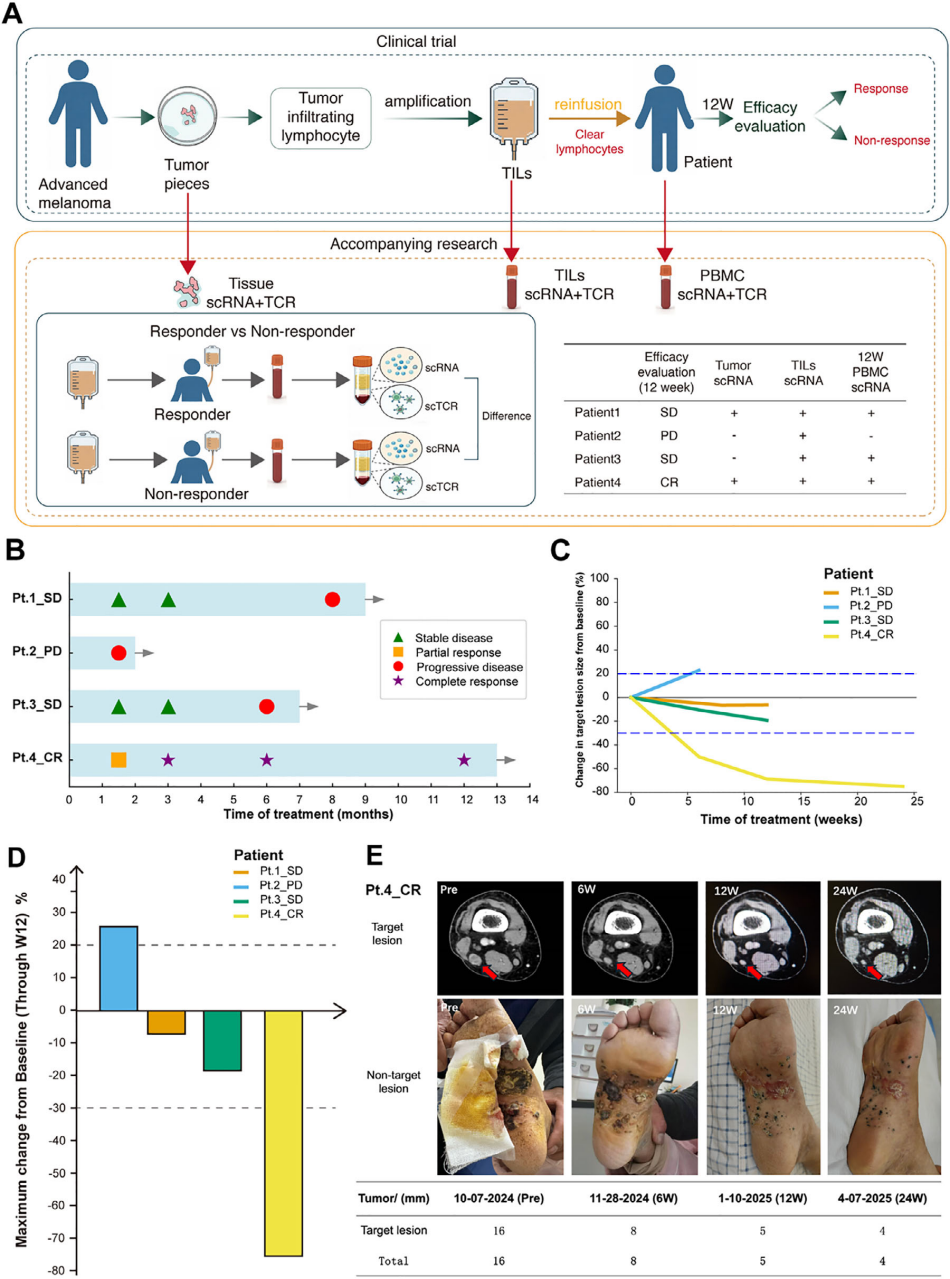
Clinical trial design and efficacy evaluation of TIL therapy in advanced melanoma. (A) Schematic overview of the clinical trial design evaluating tumor‐infiltrating lymphocyte (TIL) therapy in patients with advanced melanoma. Tumor samples were collected, expanded ex vivo to generate TILs, and reinfused into patients following lymphodepleting conditioning. Clinical evaluation was performed at baseline and at regular intervals post‐infusion. (B) Summary of clinical responses at 12 weeks post‐TIL therapy in four enrolled patients (n = 4). (C) Spider plot illustrating longitudinal changes in target lesion size over the treatment course. Each line represents an individual patient. (D) Waterfall plot showing the percentage change through 12 weeks in target lesion size from baseline according to RECIST 1.1 criteria for each patient (n = 4). (E) Representative contrast‐enhanced computed tomography and physical examination of Patient4, who achieved Complete Response (CR). The patient initially presented with recurrent plantar melanoma and lymph node metastasis; post‐TIL therapy, marked regression of both measurable and non‐measurable lesions was observed, with sustained CR maintained through 24 weeks of follow‐up.

To determine the clinical feasibility and efficacy of autologous TIL therapy in a population with a high unmet need, we initiated an Investigator‐Initiated Trial (NCT06697665) for Chinese patients with advanced acral melanoma who had progressed on prior therapies. The study integrated a comprehensive translational research component, involving single‐cell RNA and T cell receptor (TCR) sequencing of tumor tissue, the infused TIL product (LM‐103), and longitudinal peripheral blood mononuclear cells (PBMCs) to define the immunological correlates of response (Figure 1A). Four patients with unresectable stage III acral melanoma were enrolled and received a single infusion of LM‐103 following a standard non‐myeloablative lymphodepletion regimen (Baseline Clinical Characteristics are summarized in Table 1).

**TABLE 1:** Clinicopathological characteristics of patients receiving TIL therapy (n = 4).

Characteristic	Value
**Age (years)**	
Mean	56
Range	42–72
Sex, n (%)	
Male	1 (25%)
Female	3 (75%)
ECOG Performance Status, n (%)	
1	2 (50%)
2	2 (50%)
AJCC Clinical Stage, n (%)	
I	0 (0%)
II	0 (0%)
III	4 (100%)
IV	0 (0%)
Metastatic Site, n (%)	
Lymph node metastases	4 (100%)
In‐transit metastases	2 (50%)
Primary Site, n (%)	
Acral	4 (100%)
Cutaneous	0 (0%)
BRAF V600E Mutation, n (%)	
Yes	0 (0%)
No	4 (100%)
Previous Treatment, n (%)	
Anti–PD‐1 therapy	4 (100%)
Targeted therapy	3 (75%)
Chemotherapy	3 (75%)

^a^ Values are presented as the number of patients with percentage in parentheses, unless otherwise indicated. Percentages are based on total patients (n = 4). ECOG, Eastern Cooperative Oncology Group; AJCC, American Joint Committee on Cancer.

Clinical responses were assessed at 12 weeks post‐infusion. The therapy was well‐tolerated and demonstrated clear clinical activity, with a disease control rate (DCR) of 75% (3/4) and an objective response rate (ORR) of 25% (1/4) (Figure 1B). One patient (Pt. 2) experienced progressive disease (PD), two patients (Pt. 1 and Pt. 3) achieved stable disease (SD), and one patient (Pt. 4) achieved a durable complete response (CR). The heterogeneity in clinical outcomes was evident in the dynamics of tumor burden, with Pt. 4 showing a profound reduction in target lesion size of nearly 80%, while Pt. 2 exhibited rapid tumor growth (Figure 1C,D).

The clinical course of the complete responder, Pt. 4, highlights the potential for deep and lasting benefit. This patient presented with recurrent disease, including a popliteal lymph node metastasis and multiple cutaneous satellite lesions, after progressing on anti‐PD‐1 therapy. Following TIL infusion, serial contrast‐enhanced computed tomography revealed a rapid and dramatic regression of the nodal metastasis, which was classified as a partial response at 6 weeks and a complete response by 12 weeks. This radiographic response was sustained, with no evidence of disease recurrence at the one‐year follow‐up (Figure 1E). Together, these clinical data establish that autologous TIL therapy is a clinically active treatment for advanced acral melanoma, capable of mediating durable complete responses in this difficult‐to‐treat patient population.

### 2.2 Durable Response Is Associated With Persistent Systemic T Cell Dominance

**FIGURE 2:**
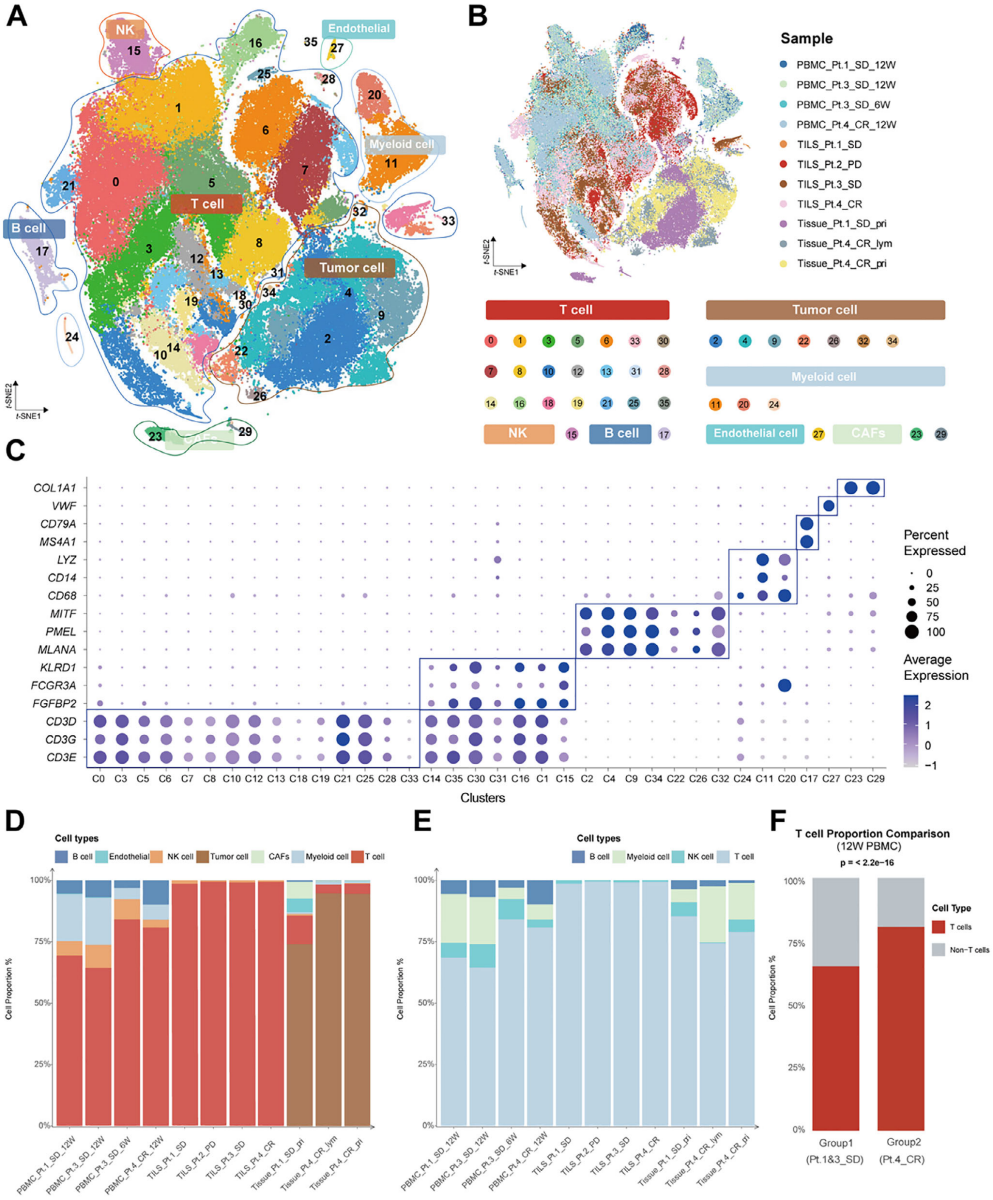
Integrated single‐cell transcriptomic landscape of tumor, TILs, and PBMCs in 4 melanoma patients receiving TIL therapy. (A) t‐SNE visualization of the integrated single‐cell RNA‐seq dataset (n = 116 629 cells) from primary tumors, metastatic lymph nodes, tumor‐infiltrating lymphocytes (TILs), and peripheral blood mononuclear cells (PBMCs) collected from four melanoma patients. A total of 36 transcriptionally distinct clusters (C0–C35) were identified based on graph‐based clustering, including T cells, tumor cells, myeloid cells, NK cells, B cells, endothelial cells, and cancer‐associated fibroblasts (CAFs). Numbers indicate individual transcriptional clusters corresponding to each lineage. (B) The t‐SNE embedding colored by sample (n = 11) origin, illustrating the distribution of cells from tumor tissues, infused TIL products, and post‐infusion PBMCs across shared cellular states. (C) Dot plot showing percent expression and average expression of 36 clusters, including 7 main cell types. Annotation of major cell lineages according to canonical marker genes, including T cells (CD3D, CD3E, CD3G), B cells (CD79A, MS4A1), Myeloid cells (CD14, LYZ), NK cells (FGFBP2, FCGR3A, KLRD1), endothelial cells (VWF), CAFs (COL1A1), and melanoma tumor cells (MITF, MLANA, PMEL). (D) Stacked bar plot depicting relative proportions of all cell types (n = 7) in each sample (n = 11). (E) Stacked bar plot depicting relative proportions of major immune cell types (n = 4) in each sample (n = 11) (F) Statistical comparison of T cell proportions in the 12 W PBMC between patients achieving complete response (CR, group2) and those with stable disease (SD, group1), revealing significantly higher T cell enrichment in the 12 W PBMC of responder group (Odds Ratio = 0.448, p < 2.2 × 10^−1^⁶, Fisher's exact test,).

To identify the immunological features associated with these divergent clinical outcomes, we performed single‐cell RNA sequencing on 11 biospecimens collected from the four patients, creating a comprehensive cellular atlas of the tumor, the therapeutic product, and the peripheral immune compartment. Unsupervised clustering of all 116 629 high‐quality cells revealed 36 distinct transcriptional states, which were annotated into major lineages including T cells, NK cells, B cells, myeloid cells, tumor cells, and stromal cells (Figure 2A–C). As expected, the infused TIL products from all four patients were highly enriched in T cells (>90%), confirming the successful ex vivo expansion of lymphocytes (Figure 2D; Table S1). In contrast, baseline tumor tissues displayed a heterogeneous mix of malignant, immune, and stromal cells, and post‐infusion PBMCs contained a mixture of circulating immune lineages (Figure 2B,E).

While baseline intratumoral T cell infiltration varied, it did not correlate with clinical outcome; for instance, Pt. 1 (SD) had the highest percentage of T cells in the primary tumor but did not achieve an objective response (Figure 2D). This observation suggests that the absolute abundance of intratumoral T cells at baseline is insufficient to predict therapeutic efficacy without consideration of T‐cell differentiation state and systemic immune dynamics. A strikingly different pattern emerged when we analyzed the peripheral blood 12 weeks after TIL infusion. The complete responder, Pt. 4, exhibited a profound and sustained T cell dominance in the circulation, with T cells comprising approximately 80% of all PBMCs (Figure 2E; Table S2). This was in stark contrast to the patients with stable disease (Pt. 1 and Pt. 3), whose peripheral T cell fractions were significantly lower. This enrichment of circulating T cells in the CR patient was highly statistically significant (*p* <2.2 × 10^−1^⁶, Fisher's exact test), suggesting that the ability to establish and maintain a systemic T cell‐biased immune environment is a key feature of a successful response (Figure 2F). These data imply that while the TIL product provides the initial antitumor effectors, it is the subsequent establishment of persistent, T cell‐dominant immunity in the peripheral circulation that correlates with durable clinical benefit.

### 2.3 TIL Product Composition Predicts Therapeutic Efficacy, With Tfh and TEX_int Cells Enriched in Responders

**FIGURE 3:**
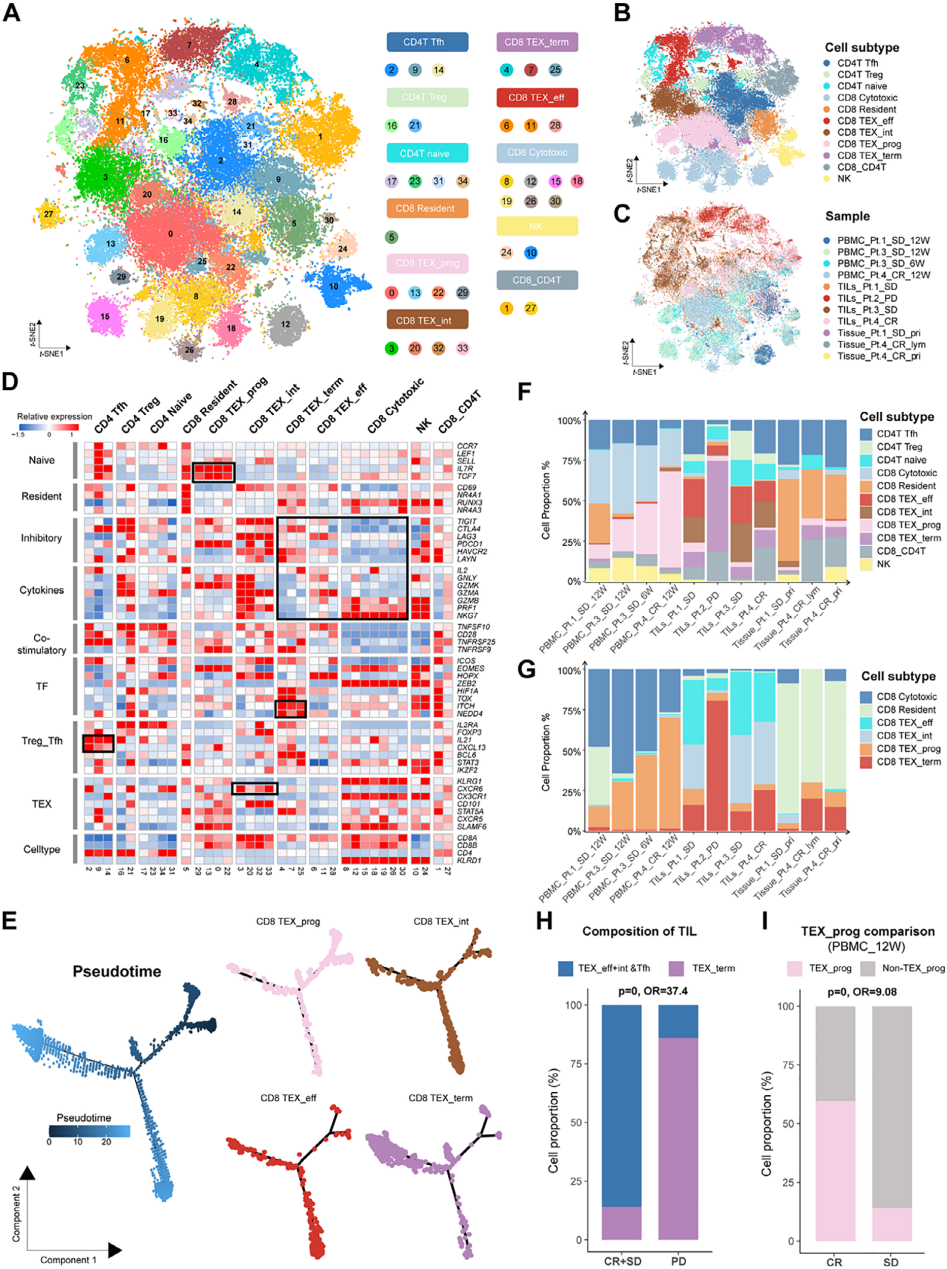
Transcriptional heterogeneity and functional states of T cells in tumor, lymphatic, and peripheral compartments following TIL therapy. (A) t‐SNE projection of all T and NK cells (n = 65414 cells) from integrated single‐cell RNA‐seq data, with clusters annotated into distinct T‐cell and NK‐cell subsets based on canonical marker gene expression. Numbers indicate individual clusters corresponding to each annotated subtype. (B,C) The same t‐SNE embedding colored by cell type (B) or sample origin (C), illustrating differences in cell‐type annotation and sample distribution across the identified clusters. (D) Heatmap indicating the expression of selected gene sets in NK & T subtypes, including naive, resident, inhibitory, cytokines, co‐stimulatory, transcriptional factors (TF), Treg & Tfh, exhausted T cells (TEX), and cell type. (E) Pseudotime‐ordered analysis of CD8+ TEX cells (n = 10 000 cells), and TEX subtypes are labeled by colors. Pseudotime trajectory analysis revealed a continuous differentiation pathway from TEX_prog → TEX_int, which subsequently bifurcates into TEX_eff or TEX_term branches, consistent with the “developmental fork” model of CD8⁺ T‐cell exhaustion, validating the functional hierarchy among TEX subsets. (F) Stacked bar plot depicting relative proportions of all NK & T subtypes in each sample. (G) Stacked bar plot depicting relative proportions of all CD8^+^ T subtypes in each sample. (H) Quantitative comparison of TIL composition between the response patients (CR and SD) and non‐response patients (PD), confirming significantly higher proportions of Tfh and TEX_int cells in responders (*p* = 0, Odds Ratio = 37.4, Fisher's exact test). (I) Quantitative comparison of CD8+ T subtypes in 12 weeks PBMCs demonstrated a significantly higher proportion of TEX_prog cells from the complete responder (CR) compared with patients with stable disease (SD) (Fisher's exact test, *p* = 0, OR = 9.08).

Having established the central role of T cells, we next sought to identify which specific T cell functional states within the infused TIL product determine therapeutic efficacy. We performed a focused re‐clustering of all T and NK cells from our dataset (n = 65414 cells), identifying multiple distinct CD4⁺ and CD8⁺ T cell subsets, including naïve, regulatory (Treg), T follicular helper (Tfh), cytotoxic, and a spectrum of exhausted T cells (TEX) ranging from progenitor (TEX_prog) to intermediate (TEX_int), effector (TEX_eff) and terminally exhausted (TEX_term) states (Figure 3A–D). In pseudo‐time analysis, we randomly selected 10000 high‐quality exhausted CD8+ T (TEX) cells to establish a pseudo‐temporal ordering reflective of cell lineage (See Methods). Further analysis of this exhaustion spectrum using pseudotime revealed that exhausted CD8^+^ T cells follow a developmental continuum from TEX_prog to TEX_int. This pathway then bifurcates into either effector‐like (TEX_eff) or terminally exhausted (TEX_term) branches, consistent with the “Divergent model” [14] of CD8^+^ T cell exhaustion (Figure 3E).

Quantitative analysis of the composition of the infused TIL products revealed a clear bifurcation between responders and non‐responder. The TIL product of the non‐responding patient (Pt. 2) was overwhelmingly dominated by terminally exhausted (TEX_term) CD8⁺ T cells, a cell state associated with poor effector function and proliferative capacity (Figure 3F; Table S3). In sharp contrast, the TIL products of the responding patients (Pt. 1, Pt. 3, and especially the complete responder Pt. 4) were significantly enriched for three key populations: CD4⁺ T follicular helper (Tfh) cells, CD8⁺ intermediate exhausted (TEX_int), and CD8⁺ effector exhausted T cells (Figure 3F; Table S3). This enrichment of functional (Tfh, TEX_eff, TEX_int) versus terminally dysfunctional (TEX_term) cells in responders was statistically significant (p = 0, Odds Ratio = 37.4, Fisher's exact test), establishing the cellular composition of the TIL product as a powerful predictive biomarker (Figure 3F,H; Table S3).

Analysis of post‐infusion PBMCs provided further insight into the mechanism of durable response. While cytotoxic T cells persisted in all patients at 12 weeks, the complete responder (Pt. 4) was uniquely distinguished by a significant enrichment of progenitor‐exhausted (TEX_prog) CD8⁺ T cells in the circulation (Figure 3G,I; Table S4). This stem‐like population is known for its capacity for self‐renewal and its ability to replenish effector T cell pools. Its presence suggests that the infused TILs in the CR patient successfully established a long‐lived, self‐renewing reservoir of antitumor T cells. Thus, the therapeutic potential of a TIL product is dictated not by the total number of T cells, but by its functional composition; enrichment of Tfh and TEX_int cells predicts initial response, while the successful engraftment and persistence of a TEX_prog reservoir in the periphery underlies durable immunity.

### 2.4 Effective TIL Products Are Defined by A Robust Tfh‐Driven Helper‐Effector Communication Network

**FIGURE 4:**
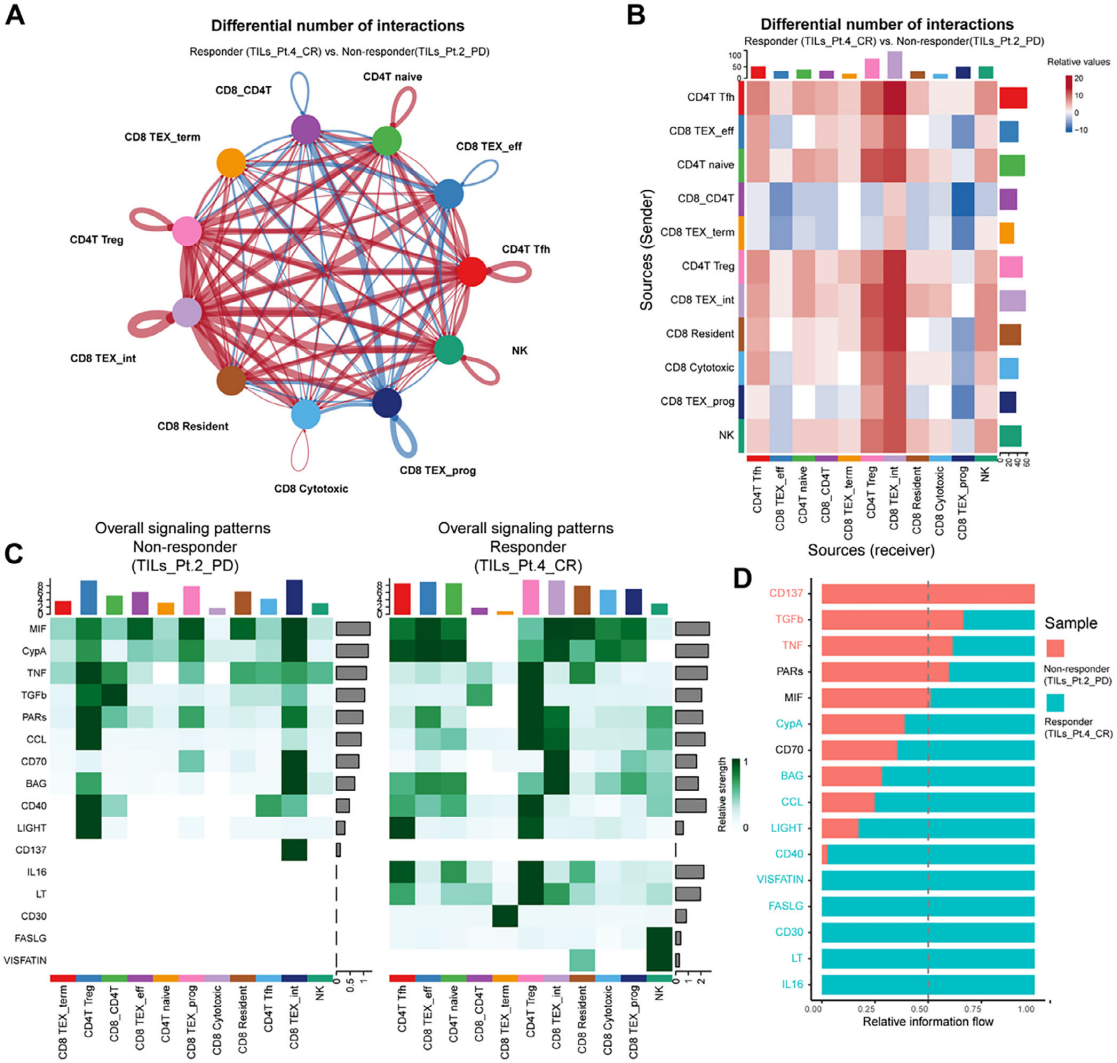
Enhanced immune cell communication networks in responder (R) compared to non‐responder (NR) melanoma patients following TIL therapy. (A) Comparison of the differential interaction number of the 11 annotated cell subtypes between Responder (Pt.4) and Non‐responder (Pt.2). The red line represents the increased interaction number, and the bule line represents decreased interaction number (B) Corresponding heatmap indicating the inferred interaction number among 11 cell subtypes between Responder (Pt.4) and Non‐responder (Pt.2). The red line represents the increased interaction number, and the bule line represents decreased interaction number (C) Heatmap shows global comparison of signaling pathway activities between Responder (Pt.4) and Non‐responder (Pt.2). The horizontal axis represents the cell type, and the vertical axis represents the signal pathway. (D) Significant signaling pathways were ranked based on their differences of overall information flow within the inferred networks between Responder and Non‐responder. The top signaling pathways colored in red are more enriched in Non‐responder, and the bottom ones colored in green were more enriched in the Responder.

Given the strong association of Tfh and TEX_int cells with clinical response, we hypothesized that the interactions between these and other T cell subsets might be critical for a coordinated antitumor attack. To investigate this, we used “CellChat” to model the intercellular communication networks within the TIL products of the complete responder (Pt. 4) and the non‐responder (Pt. 2). This analysis revealed a dramatic difference in the architecture of cellular communication. The responder's TIL product was characterized by a dense and highly interconnected network, with Tfh and TEX_int cells acting as central communication hubs (Figure 4A). In contrast, the non‐responder's network was sparse and fragmented, indicative of poor cellular coordination (Figure 4A,B).

Tfh cells emerged as the dominant signaling senders in the responder's network, while TEX_int cells were the primary signal receivers, highlighting a critical helper‐effector signaling axis (Figure 4B). Analysis of the specific signaling pathways driving these interactions further distinguished the two products. The responder network was dominated by costimulatory and pro‐inflammatory pathways essential for T cell activation, survival, and function, including CD137 (4‐1BB), CD70, CD40, and IL16 (Figure 4C,D). Conversely, the non‐responder's network was governed by immunosuppressive or dysfunctional pathways such as TGFβ and MIF. These findings demonstrate that a successful TIL product is not merely a collection of individual cells but a functional ecosystem. Effective TIL products are defined by a robust, Tfh‐driven communication network that promotes and sustains effector T cell function through coordinated costimulatory and cytokine signaling, a feature conspicuously absent in non‐responder products.

### 2.5 Durable Antitumor Immunity Is Driven by Clonal Expansion and Lineage Persistence of TEX_int Cells

To dissect the clonal mechanisms underlying durable clinical response, we integrated single‐cell transcriptomic and TCR sequencing analyses of infused TIL products and longitudinal peripheral blood samples.

**FIGURE 5:**
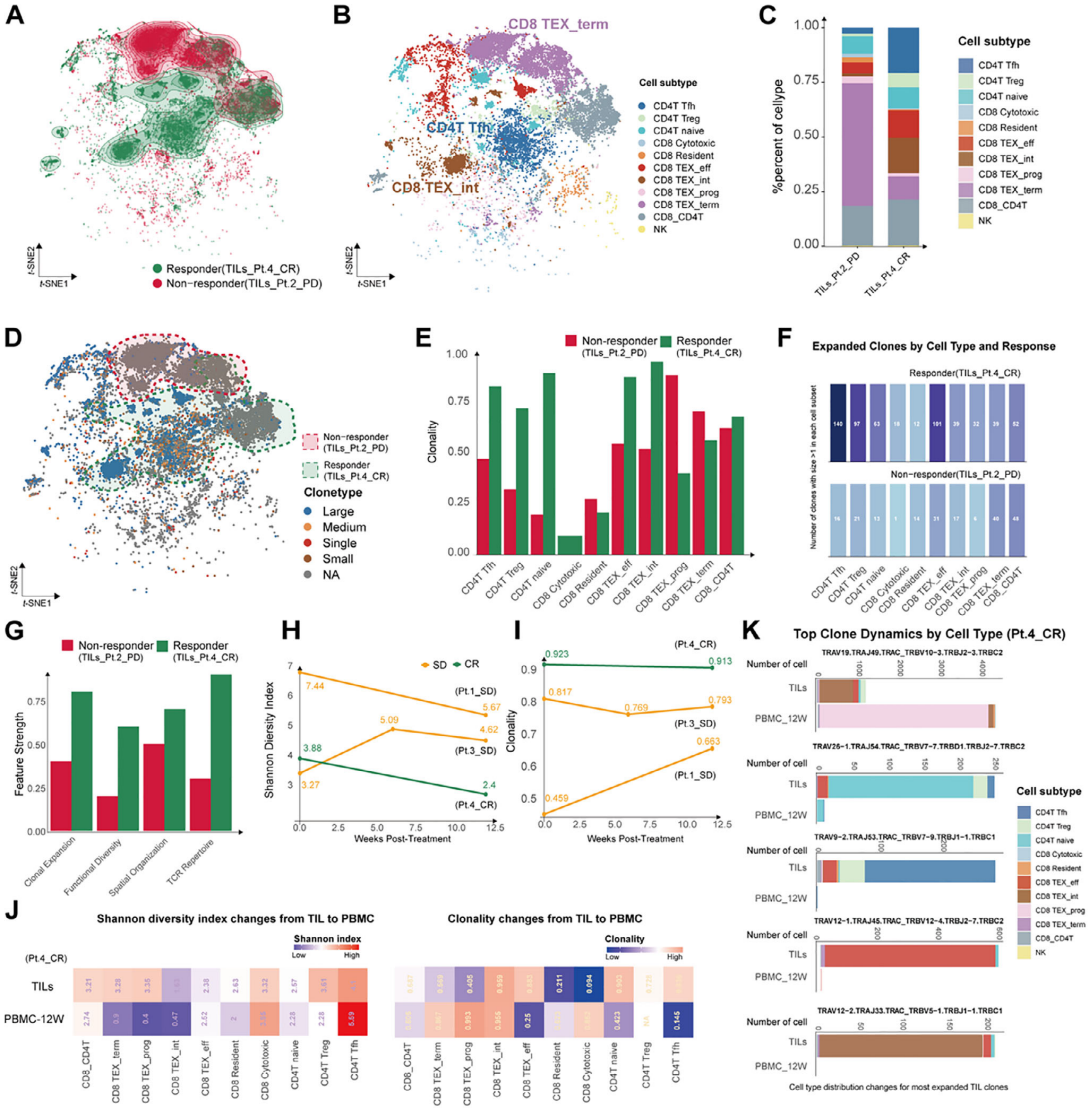
Comparative TCR Clonality and Longitudinal Repertoire Dynamics Between Responders and Non‐Responders, and Within Individual Patients Following TIL Therapy. This figure integrates comparative analyses of TCR clonality, diversity, and expanded clone distribution between responder (R) and non‐responder (NR) TIL products (Panels A–G), with longitudinal tracking of T‐cell receptor (TCR) repertoires from infused TILs to peripheral blood mononuclear cells (PBMCs) (Panels H–K). (A) t‐SNE projection of infused TIL products from the responder (Pt.4_CR) and non‐responder (Pt.2_PD), showing distinct distributions of transcriptional states between the two products. (B) The same embedding colored by annotated T‐cell and NK‐cell subtypes, highlighting CD4 Tfh, CD8 TEX_int & term subtypes. (C) Stacked bar plot showing the relative proportions of major cell subtypes within the infused TIL products of the responder and non‐responder. (D) t‐SNE projection colored by TCR clonotype size (Large, Medium, Small, Single, NA), illustrating the distribution of expanded clones across transcriptional states in responder and non‐responder TILs.(E) Comparison of TCR clonality across major cell subtypes between responder and non‐responder infused TIL products. (F) Distribution of expanded TCR clones across cell subtypes in responder and non‐responder TILs, shown as the number of cells belonging to expanded clonotypes within each subset. (G) Global repertoire features comparing responder and non‐responder TIL products, including clonal expansion, functional diversity, spatial organization, and TCR richness. (H) Longitudinal changes in TCR diversity (Shannon diversity index) from infused TILs to peripheral blood mononuclear cells (PBMCs) at 6 weeks and 12 weeks post‐infusion in Patients 1, 3, and 4. (I) Temporal dynamics of TCR clonality from infused TILs to PBMCs over time in the same patients. (J) Within‐patient analysis of Patient 4 (CR) showing changes in TCR diversity and clonality across major T‐cell subtypes from infused TILs to 12‐week PBMCs. (K) Dynamics of the most expanded TCR clones in Patient 4, showing the distribution of top clonotypes across cell subtypes in infused TILs and 12‐week PBMCs.

As an initial step, we jointly embedded T cells from responder (R) and non‐responder (NR) TIL products to establish a shared transcriptional landscape (Figure 5A). This analysis revealed a marked segregation between R and NR cells, indicating fundamentally distinct cell‐state compositions at baseline. Annotation of functional T‐cell subsets further demonstrated that responder‐derived cells were preferentially localized within T follicular helper (Tfh) and intermediate exhausted (TEX_int) compartments, whereas non‐responder cells were predominantly confined to terminally exhausted (TEX_term) states (Figure 5B). Quantitative comparison confirmed substantial differences in subset proportions between the two products (Figure 5C).

Having established these cell‐state differences, we next mapped TCR clonotypes onto the same transcriptional space to determine how clonal expansion aligned with functional identity. Strikingly, large and medium expanded clonotypes were almost exclusively derived from the responder and were concentrated within Tfh and TEX_int regions, whereas the non‐responder exhibited sparse and scattered clonal expansion (Figure 5D–G). These findings indicate that the superior quality of the responder's TIL product is encoded not only at the transcriptional level, but also at the clonal level, reflecting preferential amplification of tumor‐reactive T cells poised for helper and effector functions.

Longitudinal tracking of the TCR repertoire in the peripheral blood post‐infusion revealed the dynamics of immune persistence. While T cell diversity contracted in all patients over time, the complete responder (Pt. 4) uniquely maintained a stably high level of clonality, signifying the persistent dominance of the most effective antitumor clones (Figure 5H,I). The definitive mechanism underlying this durable immunity was revealed by tracking the fate of the most abundant clonotypes. We identified a dominant TCR clone in the CR patient that was highly enriched in the TEX_int population within the infused TIL product. Strikingly, this exact same clone was found to persist and become a major component of the self‐renewing TEX_prog population in the patient's peripheral blood 12 weeks later (Figure 5J,K).

This finding provides direct evidence of a TEX_int to TEX_prog lineage transition in vivo. It demonstrates that the intermediate exhausted T cells present in the therapeutic product are not merely transient effectors but are capable of engrafting and establishing a long‐lived, progenitor‐like T cell reservoir. Therefore, durable clinical response is driven by the infusion of clonally expanded TEX_int cells that successfully persist as a self‐renewing TEX_prog population, providing a continuous source of potent antitumor immunity.

## 3 Discussion

In this study, we demonstrate that autologous TIL therapy is a feasible and clinically active treatment for Chinese patients with advanced acral melanoma, a population with historically poor outcomes with conventional immunotherapies. In our cohort, TIL therapy achieved a 75% disease control rate and induced a durable complete response, providing a critical proof‐of‐concept for this approach in a non‐Caucasian population with a distinct melanoma subtype. More importantly, by integrating deep single‐cell and clonal analyses, we delineate a mechanistic framework that explains the biological basis of a successful and durable response. We propose a central argument that durable clinical benefit is determined by the quality of the infused TIL product, specifically the enrichment of a coordinated ecosystem of T follicular helper (Tfh) and intermediate exhausted (TEX_int) cells, which seeds a persistent, self‐renewing progenitor‐like T cell reservoir in vivo.

Our clinical findings are notable in the context of acral melanoma's well‐documented resistance to ICIs [15]. The ability to achieve a durable CR in a patient who had progressed on anti‐PD‐1 therapy underscores the potential of TILs to overcome the limitations of existing treatments. This success is likely attributable to the infusion of a broad repertoire of tumor‐reactive T cells that can recognize a diverse array of antigens, bypassing the need to reactivate a potentially limited or dysfunctional endogenous T cell pool.

The central discovery of our work is that the therapeutic potential of a TIL product is encoded in its cellular composition. We identified the Tfh and TEX_int subsets as key determinants of response. The enrichment of Tfh cells aligns with a growing body of evidence implicating these cells in robust antitumor immunity, likely through their support of CD8⁺ T cell function and their role in organizing tertiary lymphoid structures [16, 17]. Concurrently, the prevalence of TEX_int cells, a plastic and functionally competent population, contrasts sharply with the dominance of terminally exhausted T cells in the non‐responder product. This finding refines our understanding of T cell exhaustion, highlighting that an intermediate state retains therapeutic potential, whereas terminal exhaustion signifies a point of no return [18]. Together, the Tfh‐TEX_int axis represents a powerful biomarker of TIL product quality, shifting the paradigm from a simple quantitative measure of T cells to a qualitative assessment of a functional, cooperative immune network. This was further supported by our cell‐cell communication analysis, which revealed a highly active, costimulatory signaling network orchestrated by Tfh and TEX_int cells in the responder product.

Clinically validated tumor‐infiltrating lymphocyte (TIL) products, such as lifileucel (LN‐144) [11], have demonstrated meaningful clinical activity in advanced melanoma, including durable responses in a subset of heavily pretreated patients [19]. Early evaluations of TIL efficacy primarily emphasized overall CD8⁺ T‐cell abundance and total TIL expansion as surrogate markers of potency. However, accumulating evidence indicates that the differentiation state and functional composition of infused T cells are critical determinants of therapeutic outcome. Consistent with this emerging paradigm, our data show that responder TIL products are enriched for T follicular helper (Tfh) cells and intermediate exhausted (TEX_int) CD8⁺ T cells, whereas non‐response is associated with dominance of terminally exhausted subsets. The observed in vivo lineage persistence of TEX_int cells further supports the notion that qualitative features of TIL composition, rather than T‐cell quantity alone, may underlie durable clinical benefit.

Perhaps our most significant finding is the direct evidence of lineage continuity from infused TEX_int cells to a persistent, circulating TEX_prog reservoir in the complete responder. This observation provides a definitive cellular mechanism for long‐term immunological memory and durable tumor control following ACT. It suggests that successful TIL therapy is akin to a cellular transplant, where the infused product must not only mediate immediate tumor killing but also successfully engraft and establish a self‐renewing source of antitumor immunity. This model, where the therapeutic product itself seeds the long‐term memory pool, clarifies a long‐standing question in the field about the origins of sustained T cell responses post‐therapy [20].

Several limitations of this study should be acknowledged. Most notably, the cohort size is small, reflecting the investigator‐initiated and exploratory nature of this early‐phase clinical trial. Although the depth and resolution of our integrated scRNA‐seq and scTCR‐seq analyses enable robust longitudinal and clonal immune tracking, the predictive value of the Tfh/TEX_int signature and the observed lineage dynamics will require validation in larger, prospective clinical studies. In addition, paired fresh samples suitable for multiparameter flow cytometry were not available for all longitudinal time points, as all collected biospecimens were prioritized for single‐cell sequencing at the time of study execution, and repeated invasive sampling was not feasible in this clinical setting.

Nonetheless, our findings have immediate translational implications. The relative abundance of Tfh and TEX_int cells could be developed into a potency assay or release criterion for TIL manufacturing, enabling the prospective selection of more effective products. Future research should focus on optimizing ex vivo expansion protocols—perhaps through the addition of specific cytokines or small molecules—to preferentially expand these beneficial populations and prevent terminal differentiation. Furthermore, monitoring the emergence and persistence of the TEX_prog population in peripheral blood could serve as a powerful pharmacodynamic biomarker of durable response.

In conclusion, this work defines the cellular and clonal architecture of a successful TIL therapy response in acral melanoma. We demonstrate that clinical efficacy is not simply about infusing large numbers of T cells, but about transplanting a functionally integrated and clonally robust immune ecosystem. The cooperative Tfh and TEX_int cell populations within this ecosystem are the critical drivers of both immediate efficacy and, through their ability to establish a persistent progenitor reservoir, durable, life‐long immunity.

## 4 Methods

### 4.1 Ethic Approval

All clinical specimens in this study were collected with informed consent for research use and were approved by the Tianjin Medical University Cancer Hospital Institutional Review Boards in accordance with the Declaration of Helsinki, under protocol number E20231311. Consent to publish relevant clinical information potentially identifying individuals (e.g., age, gender, clinical stage, etc.) was obtained. No compensation was provided to the participants in this study.

### 4.2 Patient Enrollment and Clinical Data Collection

This clinical trial enrolled four patients with advanced melanoma (1 male, 3 females; average age 56 years, range 42–72 years) from September 2022 to February 2024. All patients had experienced disease progression after prior treatments, including immune checkpoint inhibitors, chemotherapy, and targeted therapy. Patients were treated with adoptive TIL therapy, and clinical responses were evaluated 12 weeks after TIL infusion. Clinical data, including demographic information, treatment history, Eastern Cooperative Oncology Group (ECOG) performance status, and metastatic sites, were collected and summarized in Table 1. Tumor responses and performance status were assessed per RECIST v1.1 and ECOG criteria, respectively [21, 22].

### 4.3 Clinical Trial Design and Treatment

This study was designed as an exploratory clinical trial to evaluate the safety, tolerability, and preliminary clinical efficacy of functionally enhanced autologous tumor‐infiltrating lymphocyte (TIL) therapy in patients with advanced acral melanoma. The primary objective of the study was to assess the safety and tolerability of TIL infusion. The secondary objective was to evaluate preliminary antitumor efficacy. Clinical responses were assessed according to the Response Evaluation Criteria in Solid Tumors (RECIST) version 1.1, using objective response rate (ORR) and disease control rate (DCR) as efficacy endpoints. Prior to TIL infusion, patients underwent a non‐myeloablative lymphodepletion regimen consisting of cyclophosphamide (30 mg/kg/day) administered intravenously for 2 consecutive days, followed by fludarabine (25 mg/m^2^/day) for 5 consecutive days. Autologous TILs were infused intravenously approximately 24 h after completion of lymphodepletion. Following TIL infusion, patients received systemic interleukin‐2 (IL‐2) supportive therapy to promote T‐cell survival and expansion. IL‐2 administration was initiated on day 2 after TIL infusion and delivered intravenously at a dose of 200 000 IU/kg once daily (QD) for 7–12 consecutive days, depending on patient tolerance and clinical condition. Tumor assessments were performed at 6 weeks after TIL infusion and subsequently every 6 weeks until disease progression or treatment discontinuation.

### 4.4 TILs Manufacturing

TILs were manufactured by Suzhou Lanma Biotechnology Co. according to Good Manufacturing Practice (GMP) standards procedure previously described [23]. Briefly, freshly resected tumor specimens were transported in sterile RPMI 1640 medium and mechanically dissected into 2–3 mm^3^ fragments. Tumor fragments (5–10 per well) were cultured in RPMI 1640 supplemented with 10% human AB serum and i6000 IU/mL IL‐2 (PeproTech, RRID: AB_1268861) to initiate TIL outgrowth (pre‐REP). Cultures were maintained for 10–14 days with periodic media replacement and expansion when cell density exceeded 2 × 10⁶ cells/ml. TILs generated during pre‐REP were cryopreserved and subsequently expanded using a 14‐day rapid expansion protocol (REP) prior to infusion. REP was performed using anti‐CD3 antibody (CDE‐M120a, ACROBiosystems, RRID: AB_3076268), irradiated feeder cells (irradiated peripheral monocytes from healthy donors), and IL‐2 (6,000 IU/mL), with cell splitting every 2–3 days to maintain optimal density. Upon completion of REP, TILs were harvested, formulated in infusion medium, and transported to the hospital within 12 h for intravenous administration, with infused cell doses of 4.2 × 10^1^⁰, 1.8 × 10^1^⁰, 7.2 × 10^1^⁰, and 1.2 × 10^11^ cells for Patients 1–4, respectively.

### 4.5 Clinical Efficacy Evaluation

Patients were monitored for 12 weeks post‐TIL infusion to assess clinical response. Tumor response was evaluated according to RECIST 1.1 criteria, classifying outcomes as Complete Response (CR), Partial Response (PR), Stable Disease (SD), or Progressive Disease (PD). Disease Control Rate (DCR) was defined as the proportion achieving CR, PR, or SD, and Objective Response Rate (ORR) was defined as the proportion achieving CR or PR.

### 4.6 Single Cell RNA Sample Collection and Sequencing

Tumor tissue samples (n = 3 lesions), tumor‐infiltrating lymphocyte (TIL) infusion products (n = 4), and peripheral blood mononuclear cells (PBMCs, n = 4) were collected from four patients with acral melanoma for single‐cell transcriptomic analysis. Detailed sample correspondence is shown in Figure 1A. All samples were obtained with written informed consent and approval from the Ethics Committee of Tianjin Medical University Cancer Institute and Hospital.

Processing of Tumor Tissue Samples. Fresh tumor specimens were surgically resected and immediately immersed in complete medium consisting of Dulbecco's Modified Eagle Medium (DMEM; Gibco, Thermo Fisher Scientific, Cat. No. 11054001) supplemented with 10% fetal bovine serum (FBS; Gibco, Thermo Fisher Scientific, Cat. No. 16140‐071). Samples were transported on ice to the laboratory and dissected into 1–3 mm fragments. Single‐cell suspensions were generated by mechanical dissociation followed by enzymatic digestion using collagenase type IV (Sigma–Aldrich, Cat. No. C5138) and DNase I (Roche, Cat. No. 10104159001). Cell suspensions were filtered through 40 µm cell strainers (Corning, Cat. No. 352350), washed with phosphate‐buffered saline (PBS; Gibco, Cat. No. 10010–023), and assessed for viability prior to downstream processing.

Processing of TIL Infusion Products. TIL infusion products were collected immediately prior to clinical administration. Cells were washed twice with PBS containing 0.04% bovine serum albumin (BSA; Sigma–Aldrich, Cat. No. A9418) to remove residual cytokines and culture components, followed by filtration through 40 µm cell strainers (Corning, Cat. No. 352340) to eliminate aggregates. Cell viability was assessed using trypan blue staining (0.4%; Gibco, Cat. No. 15250‐061), and only samples with viability greater than 85% were processed for single‐cell capture.

Processing of PBMC Samples. Peripheral blood samples were collected in EDTA‐coated tubes (BD Vacutainer) at designated time points. PBMCs were isolated by density gradient centrifugation using Ficoll‐Paque PLUS (Cytiva, Cat. No. 17‐1440‐02) according to the manufacturer's instructions. Isolated PBMCs were washed twice with RPMI 1640 medium (Gibco, Cat. No. 11875‐093) supplemented with 2% FBS, filtered through 40 µm strainers, and counted. Samples with viability greater than 90% were used for single‐cell library preparation.

### 4.7 Single‐Cell Library Preparation and Sequencing

Single‐cell RNA sequencing libraries were prepared using the Chromium Single Cell 5′ Reagent Kits v2 (10x Genomics), including the Chromium Single Cell 5’ Library & Gel Bead Kit (10xGenomics, PN‐1000014), following the manufacturer's protocols. Approximately 10,000 cells were loaded per channel, yielding an average recovery of ∼8000 single cells.

Following cell encapsulation, barcoded reverse transcription, cDNA amplification, fragmentation, adaptor ligation, and sample index attachment were performed using the Chromium Controller (10x Genomics). Final libraries were sequenced on an MGISEQ‐2000 platform (MGI) at BGI Genomics (Beijing, China), generating approximately 120 Gb of raw sequencing data per sample.

### 4.8 Single‐Cell TCR Sequencing and Clonotype Analysis

Single‐cell T‐cell receptor (TCR) sequencing was performed using the 10x Genomics Chromium Single Cell V(D)J platform, including the Chromium Single Cell V(D)J Enrichment Kit, Human T Cell (10xGenomics, PN‐1000005). Paired TCR α and β chain sequences were assembled using Cell Ranger VDJ (v6.1.1). Unique clonotypes were defined by identical complementarity‐determining region 3 (CDR3) amino acid sequences.

Clonal expansion was quantified by calculating the frequency of each clonotype within a given sample. TCR repertoire diversity was assessed using the Shannon diversity index. Integration of TCR and transcriptomic data enabled mapping of clonotype distributions across T‐cell subsets and tissue compartments. Clonotype overlap between tumor, TIL products, and PBMCs was evaluated using the scRepertoire [24] package (v1.11.0).

### 4.9 Quality Control and Data Processing

Sequencing data were processed using Cell Ranger (10x Genomics) for alignment to the human reference genome, barcode processing, and unique molecular identifier (UMI) counting. Downstream analyses were performed using Seurat (v4.3.2) [25] in R.

Low‐quality cells were excluded based on predefined quality control criteria, including cells with fewer than 500 detected genes or with a mitochondrial gene fraction greater than 10%. Gene expression matrices were log‐normalized and scaled prior to dimensionality reduction and clustering. Batch effects across samples were corrected using the Harmony (v1.2.0) algorithm implemented in Seurat, following established single‐cell integration workflows. Only cells and samples that passed quality control were included in downstream analyses, and no additional outlier removal was performed beyond the stated criteria.

### 4.10 Clustering and Cell Type Annotation

After integration, cells were clustered using graph‐based clustering implemented in Seurat and visualized using 2D embeddings. Cell identities were assigned based on canonical marker gene expression, including CD3D, CD3E, and CD3G for T cells; CD79A for B cells; COL1A1 for cancer‐associated fibroblasts; and MITF for melanoma cells. Cell‐type annotations were manually curated and validated across samples. Relative cell‐type proportions were calculated for each sample and tissue compartment.

### 4.11 T‐Cell Subset and Exhaustion State Analysis

All T‐cell populations from tumor tissues, tumor‐infiltrating lymphocyte (TIL) products, and peripheral blood mononuclear cells (PBMCs) were extracted and re‐clustered. T‐cell subsets were defined based on established transcriptional signatures distinguishing CD4⁺ and CD8⁺ lineages and functional states, including naïve, effector, tissue‐resident, and exhausted populations.

Exhausted CD8⁺ T cells were further classified into progenitor exhausted (TEX_prog), intermediate exhausted (TEX_int), effector exhausted (TEX_eff), and terminally exhausted (TEX_term) states according to differentiation‐associated gene expression patterns, consistent with previously described exhaustion hierarchies [14].

### 4.12 Cell Developmental Trajectory

The cell lineage trajectory of 4 exhausted CD8+ T (TEX) subtypes was inferred by using Monocle2 [26]. First, we used the “relative2abs” function in Monocle2 to convert TPM into normalized mRNA counts and created an object with parameter “expressionFamily = negbinomial.size” following the Monocle2 tutorial. We used the “differentialGeneTest” function to derive DEG from each cluster, and genes with a *q*‐value <1e‐5 were used to order the cells in pseudotime analysis. After the cell trajectories were constructed, differentially expressed genes along the pseudotime were detected using the “differentialGeneTest” function.

### 4.13 Cell–Cell Communication Analysis

Cell–cell communication analysis was performed using the CellChat [27] package (v2.1.1) to infer intercellular signaling networks. Normalized gene expression matrices and annotated cell‐type metadata were used as inputs. CellChat identified potential ligand–receptor interactions based on the integrated human ligand–receptor database.

Interaction probability was computed for each ligand–receptor pair, and signaling networks were constructed for responders (R) and non‐responders (NR) separately. The total number and strength of inferred interactions were compared between groups. Differential pathway analysis identified signaling axes enriched in each group, with a focus on immune activation‐related pathways, including IL‐16, CD40, CD70, and FASLG. Network visualization and centrality analyses were performed to identify dominant sender and receiver cell types. Information flow was calculated to quantify the overall communication strength of each pathway.

### 4.14 Single‐Cell TCR Sequencing and Clonotype Analysis

Single‐cell T‐cell receptor (TCR) sequencing was performed using the 10x Genomics Chromium Single Cell V(D)J platform. Paired TCR α and β chain sequences were assembled using Cell Ranger VDJ (v6.1.1). Unique clonotypes were defined by identical complementarity‐determining region 3 (CDR3) amino acid sequences.

Clonal expansion was quantified by calculating the frequency of each clonotype within a given sample. TCR repertoire diversity was assessed using the Shannon diversity index. Integration of TCR and transcriptomic data enabled mapping of clonotype distributions across T‐cell subsets and tissue compartments. Clonotype overlap between tumor, TIL products, and PBMCs was evaluated using the scRepertoire [24] package (v1.11.0).

### 4.15 Statistical Analysis

Given the exploratory nature of this investigator‐initiated study and the limited cohort size, no formal sample size calculation was performed. All samples that met predefined quality control criteria were included in the analyses.

For single‐cell RNA sequencing analyses, statistical comparisons were performed on data that passed quality control and batch correction as described above. Cell‐type proportions were calculated as percentages of total cells within each sample or tissue compartment. Comparisons of cellular composition between responder and non‐responder groups were conducted using two‐sided Fisher's exact tests.

For T‐cell receptor repertoire analyses, clonality and diversity metrics were calculated at the sample level. Clonal expansion was assessed based on clonotype frequencies, and repertoire diversity was quantified using the Shannon diversity index. Statistical comparisons of clonotype‐related metrics were performed using non‐parametric tests, as appropriate.

All statistical tests were two‐sided, and a *p*‐value <0.05 was considered statistically significant. Statistical analyses were used to support descriptive and comparative interpretations of cellular composition and clonal dynamics rather than population‐level inference.

All analyses and data visualizations were performed using R (v4.3.2) with Seurat (v4.4.0), Harmony (v1.2.0), CellChat (v2.1.1), Monocle2 (v2.30.0), and scRepertoire (v1.11.0).

## Author Contributions

C.Z., W.X., and H.S. carried out the data analysis. T.L., F.L., H.L., Z.G., and H.W. were responsible for clinical efficacy evaluation, data collection, and visualization. K.C., X.L., J.Y. supervised the work.

## Conflicts of Interest

The authors declare no conflicts of interest.

## Supporting information




**Supporting File**: advs73622‐sup‐0001‐SuppMat.docx.

**Supporting File**: advs73622‐sup‐0001‐Tables 1–4.xlsx.

## Data Availability

The raw single‐cell RNA‐seq and TCR‐seq data generated in this study have been deposited in the Sequence Read Archive (SRA) under accession code PRJNA1381235 and will be publicly released upon acceptance and publication of this manuscript.
