# Supplementary material for: Single‐Cell Dissection of Tumor‐Infiltrating Lymphocytes Reveals Cellular Architecture Predictive of Therapeutic Efficacy in Acral Melanoma
Source: Adv Sci (Weinh). 2026 Jan 4;13(35):e21555. doi: 10.1002/advs.202521555 (PMC13292159; doi:10.1002/advs.202521555)
Supplement: Supplementary file 1 — Supporting File: advs73622‐sup‐0001‐SuppMat.docx. [file ADVS-13-e21555-s001.docx]

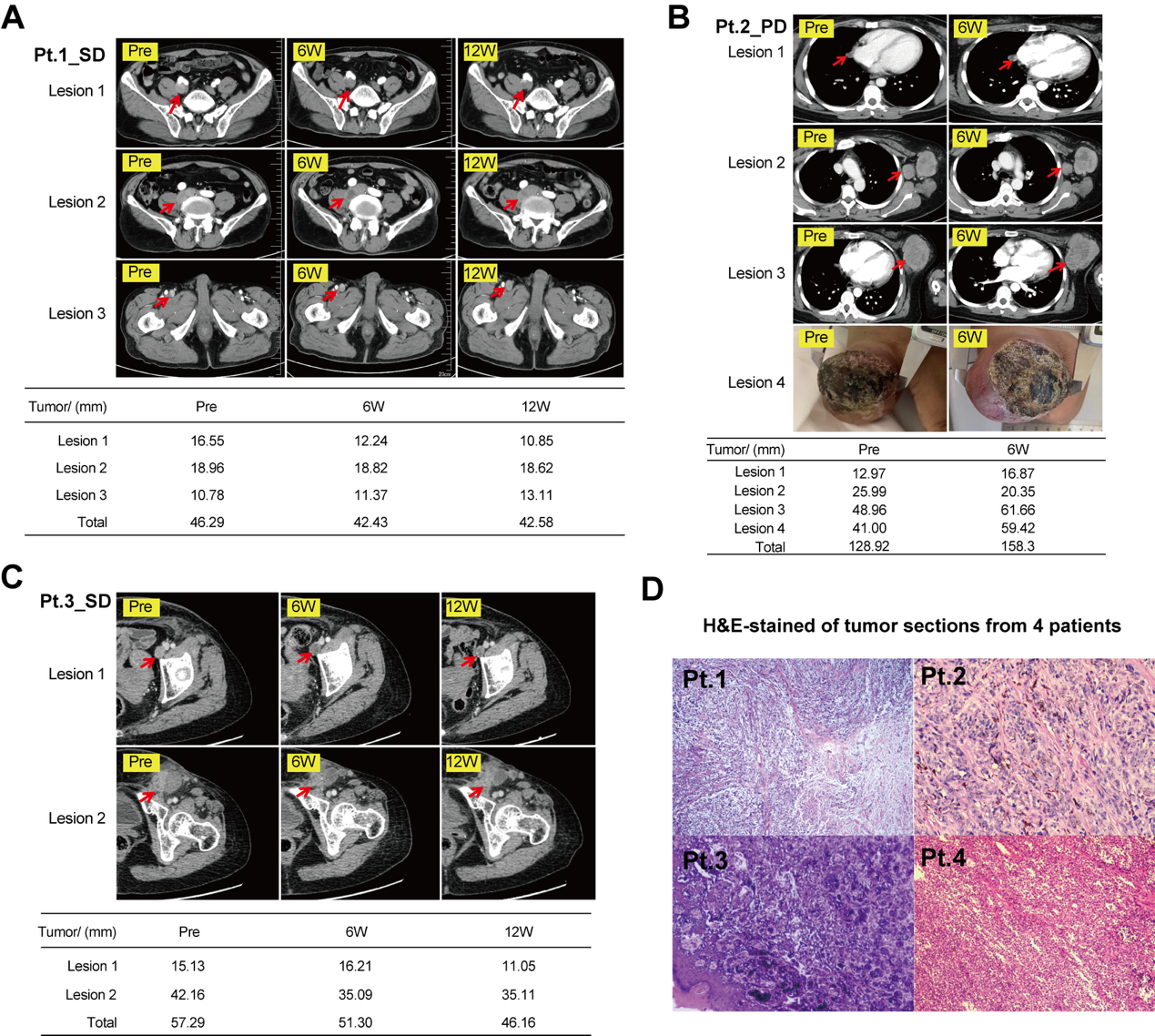


**Supplementary Figure 1. Clinical imaging and histopathological assessment following TIL therapy.**

(A) Representative contrast-enhanced computed tomography (CT) images of Patient 1 showing three target lesions at baseline (Pre), 6 weeks (6W), and 12 weeks (12W) after TIL infusion, consistent with stable disease (SD).

(B) Contrast-enhanced CT images and representative clinical photographs of Patient 2 demonstrating progressive disease (PD) with increasing tumor burden following TIL therapy.

(C) Contrast-enhanced CT images of Patient 3 showing two target lesions with an overall reduction in tumor size by 12 weeks after infusion, consistent with stable disease (SD).

(D) Representative hematoxylin and eosin (H&E)–stained tumor sections from all four patients at baseline, illustrating histopathological features of acral melanoma prior to TIL therapy.

Red arrows indicate target lesions. Tumor measurements (mm) were evaluated according to RECIST version 1.1 criteria. Pre, baseline prior to TIL infusion; 6W, 6 weeks post-infusion; 12W, 12 weeks post-infusion.
